# Supplementary material for: MetaRibo-Seq measures translation in microbiomes
Source: Nat Commun. 2020 Jun 29;11:3268. doi: 10.1038/s41467-020-17081-z (PMC7324362; doi:10.1038/s41467-020-17081-z)
Supplement: Supplementary file 10 — Supplementary Data 7 [file 41467_2020_17081_MOESM10_ESM.zip › File2/Confidence_High_Taxonomy/332722_out.krona.html]

Javascript must be enabled to view this page.

members
magnitude
magnitudeUnassigned
count
unassigned
taxon
rank

332722\_out

254

superkingdom
2
254

976
phylum
254

254
200643
class

order
171549
254

171552
family
2

1
genus
838

1

SRS018888\_contig\_number\_2707
species
165179

1
577309
genus

1
1263095
species

SRS017701\_contig\_number\_3294

10
171551
family

genus
836
10


SRS014979\_contig\_number\_1645SRS015264\_contig\_number\_10192SRS017745\_contig\_number\_contig-100\_4652.4653SRS017916\_contig\_number\_contig-100\_13106.50127SRS019808\_contig\_number\_contig-100\_18145.61859SRS056537\_contig\_number\_contig-100\_12748.142904SRS074586\_contig\_number\_contig-100\_5422.46195SRS077086\_contig\_number\_10962SRS104975\_contig\_number\_19711SRS148721\_contig\_number\_55562
658663
species
10

20
family
815

genus

SRS024435\_contig\_number\_30329SRS045645\_contig\_number\_25147SRS143876\_contig\_number\_543SRS893380\_contig\_number\_contig-100\_6.7SRS893382\_contig\_number\_4557
5
20
816


SRS043667\_contig\_number\_1342
1262750
species
1


SRS017521\_contig\_number\_42552
371601
species
1

818
species

SRS053649\_contig\_number\_10141
1

5
817
species

SRS015190\_contig\_number\_3154SRS018541\_contig\_number\_3015SRS077335\_contig\_number\_3287SRS077730\_contig\_number\_2241SRS098717\_contig\_number\_18999

1

SRS018623\_contig\_number\_6503
329854
species


SRS144362\_contig\_number\_27898
47678
species
1

3

SRS043411\_contig\_number\_12132SRS1041129\_contig\_number\_14043SRS147377\_contig\_number\_4118
821
species

1
species
820

SRS058723\_contig\_number\_6043


SRS019178\_contig\_number\_425
46506
species
1

2005525
family
220

7
220
375288
genus

SRS048981\_contig\_number\_contig-100\_5058.5059SRS064232\_contig\_number\_contig-100\_3911.3912SRS077294\_contig\_number\_8279SRS142890\_contig\_number\_37493SRS144537\_contig\_number\_14663SRS149075\_contig\_number\_7475SRS893341\_contig\_number\_7379

1
species
46503

SRS147271\_contig\_number\_29935


SRS023715\_contig\_number\_23759
species
2293115
1

469591
species

SRS893383\_contig\_number\_35388
1

1
1947048
species

SRS011084\_contig\_number\_26384

2293116
species

SRS013158\_contig\_number\_1763SRS1041133\_contig\_number\_25493
2


SRS019496\_contig\_number\_12444SRS050925\_contig\_number\_25963SRS104693\_contig\_number\_contig-100\_466.92140
species
1262912
3

2
species
658662

SRS015794\_contig\_number\_contig-100\_565.90399SRS045713\_contig\_number\_contig-100\_617.123847

199

SRS011134\_contig\_number\_2069SRS011239\_contig\_number\_18092SRS012273\_contig\_number\_45478SRS012969\_contig\_number\_32707SRS013098\_contig\_number\_46518SRS013800\_contig\_number\_23632SRS014235\_contig\_number\_14635SRS014287\_contig\_number\_15815SRS014313\_contig\_number\_1637SRS014412\_contig\_number\_17001SRS014459\_contig\_number\_23661SRS014948\_contig\_number\_6275SRS015065\_contig\_number\_19199SRS015217\_contig\_number\_28964SRS015431\_contig\_number\_contig-100\_1073.493421SRS015579\_contig\_number\_contig-100\_463.154974SRS015663\_contig\_number\_contig-100\_1048.209506SRS015782\_contig\_number\_34830SRS016056\_contig\_number\_5523SRS016095\_contig\_number\_13715SRS016267\_contig\_number\_11594SRS016335\_contig\_number\_36937SRS016629\_contig\_number\_3156SRS016989\_contig\_number\_12529SRS017103\_contig\_number\_31821SRS017191\_contig\_number\_20292SRS017433\_contig\_number\_15433SRS018313\_contig\_number\_8768SRS018351\_contig\_number\_15471SRS018575\_contig\_number\_contig-100\_905.67115SRS018656\_contig\_number\_9423SRS018836\_contig\_number\_27688SRS019030\_contig\_number\_contig-100\_5102.77028SRS019068\_contig\_number\_37481SRS019161\_contig\_number\_31710SRS019267\_contig\_number\_6183SRS019397\_contig\_number\_9061SRS019445\_contig\_number\_contig-100\_1249.185937SRS019601\_contig\_number\_contig-100\_1103.158076SRS019685\_contig\_number\_contig-100\_812.210636SRS019787\_contig\_number\_8458SRS019968\_contig\_number\_contig-100\_1218.105212SRS020233\_contig\_number\_45309SRS020328\_contig\_number\_28362SRS020394\_contig\_number\_contig-100\_1122.111220SRS020622\_contig\_number\_6843SRS021219\_contig\_number\_13050SRS021948\_contig\_number\_29004SRS022071\_contig\_number\_35678SRS022609\_contig\_number\_51182SRS022713\_contig\_number\_contig-100\_1829.59480SRS023583\_contig\_number\_contig-100\_940.106555SRS023971\_contig\_number\_1578SRS024132\_contig\_number\_37996SRS024388\_contig\_number\_contig-100\_1229.95493SRS043701\_contig\_number\_10400SRS043768\_contig\_number\_18255SRS044535\_contig\_number\_14561SRS045004\_contig\_number\_32094SRS045195\_contig\_number\_6316SRS045244\_contig\_number\_7395SRS045826\_contig\_number\_12626SRS046369\_contig\_number\_11171SRS046712\_contig\_number\_contig-100\_1389.30077SRS047014\_contig\_number\_27248SRS048060\_contig\_number\_2937SRS048870\_contig\_number\_14193SRS049773\_contig\_number\_28480SRS049896\_contig\_number\_5454SRS049900\_contig\_number\_9541SRS049959\_contig\_number\_contig-100\_1356.89873SRS050026\_contig\_number\_5179SRS050299\_contig\_number\_contig-100\_1344.78681SRS050422\_contig\_number\_35021SRS050752\_contig\_number\_29521SRS050941\_contig\_number\_15113SRS051610\_contig\_number\_9628SRS051882\_contig\_number\_contig-100\_81.129243SRS052027\_contig\_number\_16105SRS052078\_contig\_number\_394SRS052697\_contig\_number\_46291SRS053214\_contig\_number\_9424SRS053398\_contig\_number\_22824SRS053573\_contig\_number\_2310SRS054352\_contig\_number\_contig-100\_1232.99369SRS054590\_contig\_number\_344SRS056259\_contig\_number\_26233SRS056273\_contig\_number\_26436SRS056519\_contig\_number\_11280SRS057478\_contig\_number\_4049SRS058070\_contig\_number\_4396SRS058145\_contig\_number\_3809SRS058770\_contig\_number\_22343SRS062701\_contig\_number\_7321SRS063040\_contig\_number\_contig-100\_516.245219SRS063370\_contig\_number\_contig-100\_1484.77634SRS064557\_contig\_number\_11149SRS064645\_contig\_number\_196SRS064973\_contig\_number\_contig-100\_1353.17988SRS065397\_contig\_number\_13440SRS065504\_contig\_number\_32814SRS074964\_contig\_number\_contig-100\_26.57670SRS075021\_contig\_number\_3075SRS075398\_contig\_number\_598SRS075773\_contig\_number\_38905SRS075821\_contig\_number\_8702SRS075878\_contig\_number\_18376SRS076804\_contig\_number\_12238SRS076976\_contig\_number\_7795SRS077127\_contig\_number\_4827SRS077194\_contig\_number\_26089SRS077231\_contig\_number\_14400SRS077392\_contig\_number\_41868SRS077454\_contig\_number\_16249SRS077589\_contig\_number\_contig-100\_717.31610SRS077641\_contig\_number\_13843SRS077849\_contig\_number\_28273SRS078176\_contig\_number\_20552SRS078242\_contig\_number\_23978SRS078419\_contig\_number\_21641SRS078665\_contig\_number\_contig-100\_878.55433SRS097920\_contig\_number\_26155SRS097958\_contig\_number\_24170SRS098061\_contig\_number\_8536SRS098571\_contig\_number\_29184SRS098655\_contig\_number\_2445SRS100021\_contig\_number\_11859SRS103987\_contig\_number\_37330SRS104036\_contig\_number\_6763SRS1041036\_contig\_number\_contig-100\_535.42214SRS1041038\_contig\_number\_contig-100\_1297.96732SRS1041039\_contig\_number\_29762SRS1041090\_contig\_number\_3818SRS1041091\_contig\_number\_contig-100\_770.125797SRS1041092\_contig\_number\_10485SRS1041095\_contig\_number\_2735SRS1041132\_contig\_number\_contig-100\_1044.63025SRS1041134\_contig\_number\_10883SRS1041137\_contig\_number\_27593SRS1041138\_contig\_number\_10836SRS1041141\_contig\_number\_8261SRS1041142\_contig\_number\_6399SRS1041144\_contig\_number\_contig-100\_943.34545SRS1041147\_contig\_number\_858SRS104311\_contig\_number\_21462SRS104327\_contig\_number\_6065SRS1055038\_contig\_number\_1753SRS1055043\_contig\_number\_19822SRS1055049\_contig\_number\_4358SRS1055069\_contig\_number\_contig-100\_1326.96923SRS1055076\_contig\_number\_12257SRS1055095\_contig\_number\_contig-100\_2025.2026SRS1055099\_contig\_number\_2511SRS140492\_contig\_number\_6034SRS142503\_contig\_number\_45738SRS142599\_contig\_number\_17613SRS142712\_contig\_number\_3888SRS142980\_contig\_number\_contig-100\_242.243SRS143085\_contig\_number\_3167SRS143148\_contig\_number\_26728SRS143342\_contig\_number\_2230SRS143372\_contig\_number\_2741SRS143523\_contig\_number\_20912SRS143895\_contig\_number\_42586SRS144135\_contig\_number\_23867SRS144183\_contig\_number\_33075SRS144297\_contig\_number\_8761SRS144506\_contig\_number\_contig-100\_1228.290011SRS144603\_contig\_number\_contig-100\_138.109961SRS144714\_contig\_number\_9812SRS145497\_contig\_number\_12172SRS146764\_contig\_number\_27678SRS146813\_contig\_number\_6476SRS147039\_contig\_number\_contig-100\_3693.3694SRS147272\_contig\_number\_312SRS147614\_contig\_number\_2725SRS148196\_contig\_number\_35764SRS148253\_contig\_number\_8339SRS148511\_contig\_number\_11061SRS148784\_contig\_number\_19505SRS148817\_contig\_number\_8980SRS148874\_contig\_number\_11209SRS148970\_contig\_number\_3556SRS149181\_contig\_number\_28818SRS149244\_contig\_number\_9911SRS150029\_contig\_number\_10002SRS893187\_contig\_number\_10076SRS893230\_contig\_number\_10843SRS893231\_contig\_number\_1227SRS893252\_contig\_number\_17029SRS893253\_contig\_number\_120SRS893256\_contig\_number\_1573SRS893259\_contig\_number\_3424SRS893285\_contig\_number\_3787SRS893288\_contig\_number\_3854SRS893342\_contig\_number\_9534SRS893366\_contig\_number\_1360SRS971275\_contig\_number\_45674SRS971276\_contig\_number\_12612
823
species


SRS023526\_contig\_number\_20249SRS023829\_contig\_number\_contig-100\_631.15054SRS1041140\_contig\_number\_12201
species
2293114
3

family
1853231
2

1
genus
574697

544645
species

SRS075078\_contig\_number\_24633
1


SRS147346\_contig\_number\_49522
283168
genus
1
